# PPARs, Obesity, and Inflammation

**DOI:** 10.1155/2007/95974

**Published:** 2006-12-28

**Authors:** Rinke Stienstra, Caroline Duval, Michael Müller, Sander Kersten

**Affiliations:** Nutrition, Metabolism and Genomics Group and Nutrigenomics Consortium, Wageningen University, P.O. Box 8129, 6700 EV Wageningen, The Netherlands

## Abstract

The worldwide prevalence of obesity and related metabolic disorders is rising rapidly, increasing the burden on our healthcare system. Obesity is often accompanied by excess fat storage in tissues other than adipose tissue, including liver and skeletal muscle, which may lead to local insulin resistance and may stimulate inflammation, as in steatohepatitis. In addition, obesity changes the morphology and composition of adipose tissue, leading to changes in protein production and secretion. Some of these secreted proteins, including several proinflammatory mediators, may be produced by macrophages resident in the adipose tissue. The changes in inflammatory status of adipose tissue and liver with obesity feed a growing recognition that obesity represents a state of chronic low-level inflammation. Various molecular mechanisms have been implicated in obesity-induced inflammation, some of which are modulated by the peroxisome proliferator-activated receptors (PPARs). PPARs are ligand-activated transcription factors involved in the regulation of numerous biological processes, including lipid and glucose metabolism, and overall energy homeostasis. Importantly, PPARs also modulate the inflammatory response, which makes them an interesting therapeutic target to mitigate obesity-induced inflammation and its consequences. This review will address the role of PPARs in obesity-induced inflammation specifically in adipose tissue, liver, and the vascular wall.

## 1. INTRODUCTION

The prevalence of obesity worldwide has progressively increased over the past decades. In 2000, it was estimated that more than half of US adults were overweight, while the frequency of obesity, which is defined by a body mass index (BMI) ≥ 30 kg/m^2^, was 20%, reflecting an increase of 61% within 10 years [1]. Not only have more and more adults become obese, obesity is also striking at a much younger age leading to a high number of obese children and adolescents [2]. Unless drastic action is taken, many countries will face a decline in life expectancy in the 21st century due to the obesity epidemic.

Obesity is the direct result of an imbalance between energy intake and energy expenditure. The excess energy is primarily stored in adipose tissue in the form of triglycerides. Although adipocytes are specifically designed to store energy and easily fill up with fat, the morphological changes associated with adipose tissue growth are not without consequences for the organism as a whole [3]. Evidence has accumulated suggesting that in response to adipocyte hypertrophy during development of obesity, adipose tissue function is compromised.

Obesity also provokes structural and metabolic alterations in other organs, including skeletal muscle and liver. Indeed, obesity is closely linked to fat storage in liver and is nowadays considered as a major risk factor for the development of fatty liver diseases. The incidence of nonalcoholic fatty liver disorders (NAFLDs) and obesity are therefore intimately linked. It has been estimated that about 75% of obese subjects have NAFLD while 20% develop nonalcoholic steatohepatitis (NASH), which is defined as fatty liver disease with inflammation [4]. The amount of fat stored in liver is determined by the balance between fatty acid uptake, endogenous fatty acid synthesis, triglyceride synthesis, fatty acid oxidation, and triglyceride export. Changes in any of these parameters can affect the amount of fat stored in liver.

The excessive fat accumulation in adipose tissue, liver, and other organs strongly predisposes to the development of metabolic changes that increase overall morbidity risk. The metabolic abnormalities that often accompany obesity include hypertension, impaired glucose tolerance, insulin resistance leading to hyperinsulinemia, and dyslipidemia. Collectively, these abnormalities have been clustered into the metabolic syndrome or Syndrome X [5]. Individuals that are diagnosed with metabolic syndrome have a significantly increased risk of developing cardiovascular disease (CVD) and type II diabetes. Inasmuch as CVD is the major cause of death in industrialized countries, effective strategies to curtail the number of individuals with metabolic syndrome are badly needed. Visceral obesity, which is characterized by excess fat storage in and around the abdomen, is the prime cause of the metabolic abnormalities, and therefore represents an important target in the treatment of metabolic syndrome [6].

In recent years, it has become clear that obesity also gives rise to a heightened state of inflammation. The link between obesity and inflammation was first established by Hotamisligil et al. who showed a positive correlation between adipose mass and expression of the proinflammatory gene tumor necrosis factor-*α* (TNF*α*) [7]. The link between obesity and inflammation has been further illustrated by the increased plasma levels of several proinflammatory markers including cytokines and acute phase proteins like C-reactive protein (CRP) in obese individuals [8, 9]. Nowadays, CRP is considered as an independent biomarker for the development of CVD [10] which emphasizes the connection between inflammation, obesity, and CVD. Many of the inflammatory markers found in plasma of obese individuals appear to originate from adipose tissue [8]. These observations have led to the view that obesity is a state of chronic low-grade inflammation that is initiated by morphological changes in the adipose tissue.

One consequence of the elevated inflammatory status is insulin resistance. Proinflammatory cytokines originating from fat have been shown to directly interfere with insulin signaling pathways [11]. For example, TNF*α* causes insulin resistance by inhibiting tyrosine phosphorylation of insulin receptor substrate-1 (IRS-1) [12]. Other mechanisms of inhibition of IRS-1 phosphorylation by inflammatory mediators include chronic activation of JNK, PKC, and IKK [13–15].

Besides TNF*α*, adipose tissue produces a host of other adipokines with well-described effects on metabolism and inflammation. Resistin, adiponectin, leptin, and monocyte chemoattractant protein-1 (MCP-1) are among a group of secreted proteins from adipose tissue with immune-modulating functions [16]. The production and secretion of these adipokines are altered during obesity, resulting in a more proinflammatory or atherogenic secretion profile. Indeed, whereas secretion of MCP-1, resistin, and other proinflammatory cytokines is increased by obesity, the adipose secretion of the anti-inflammatory protein adiponectin is decreased [17].

Although increased visceral fat depots [6] and adipocyte hypertrophy [3] had been linked to a higher degree of adipose inflammation, until recently the exact pathways leading to a proinflammatory state of adipose tissue in obese individuals remained unidentified. However, recently much attention has been diverted to the role of macrophages. In 2003, two papers published back to back showed that diet-induced obesity is associated with infiltration of macrophages into white adipose tissue [18, 19]. Infiltrated macrophages, which are part of the stromal vascular fraction of adipose tissue, are subsequently responsible for the production of a wide variety of proinflammatory proteins including MCP-1, TNF*α*, and interleukin-6 (IL-6). The development of insulin resistance in adipocytes was closely linked to the infiltration of macrophages. However, if and how entry of macrophages into white adipose tissue (WAT) leads to systemic insulin resistance remains unclear, although it is increasingly believed that altered secretion of adipokines by WAT during obesity may represent an important piece of the puzzle.

One of the other tissues that is affected by the enlargement and proinflammatory secretion profile of adipose tissue is the liver. Chronic activation of the master regulator of inflammation nuclear factor-*κ*B (NF-*κ*B) by cytokines has been directly linked to the development of insulin resistance in liver [20, 21]. It has also been shown that adipose-specific overexpression of MCP-1 increases hepatic triyglyceride content [22]. Although steatosis is a common occurrence in obese individuals, the role of inflamed adipose tissue in development of steatosis needs further exploration.

Initially characterized by excess fat storage, steatosis can progress to steatohepatitis and finally leads to cirrhosis and structural alterations of the liver [23]. The molecular mechanisms underlying the development of steatosis and progression to steatohepatitis remain poorly understood. Whereas some patients only develop steatosis, others develop steatohepatitis and fibrosis. Lipid peroxidation, cytokines, and other proinflammatory compounds are believed to play a vital role in the transition [4]. In addition, the role of the expanding adipose tissue might also prove relevant to the development of steatohepatitis.

Recently, the elevated inflammatory status of adipose tissue and concurrent increased production of adipose tissue-derived cytokines have also been connected with atherosclerosis. Initially defined as a pathological lipid deposition, the atherosclerotic process is nowadays considered as an ongoing inflammatory process in which numerous cytokines, chemokines, and inflammatory cells participate [24]. Independent of its connection to the metabolic syndrome, obesity itself is a known risk factor for the development of atherosclerosis and CVD [25].

In summary, obesity represents a major health threat, and effective therapies to minimize obesity-related comorbidities are sorely needed. By targeting the inflammatory component, the progression of obesity towards insulin resistance and CVD might be slowed down.

The ligand-activated transcription factors belonging to the peroxisome proliferators- activated receptor (PPAR) family are involved in the regulation of inflammation and energy homestasis and represent important targets for obesity, obesity-induced inflammation, and metabolic syndrome in general. These receptors share a common mode of action that involves heterodimerization with the nuclear receptor RXR and subsequent binding to specific DNA-response elements in the promoter of target genes. Binding of ligands to PPARs leads to recruitment of coactivators and chromatin remodeling, resulting in initiation of DNA transcription [26, 27]. Currently, synthetic PPAR agonists are widely used for the treatment of insulin resistance and dyslipidemia. This review will explore the role of PPARs in governing chronic inflammation with special emphasis on the connection with metabolic syndrome. The link with obesity and inflammation will be discussed separately for the three PPAR isoforms: PPAR*α*, PPAR*β*/*δ*, and PPAR*γ*.

## 2. PPAR*α*

PPAR*α* is well expressed in metabolically active tissues including liver, brown adipose tissue, muscle, and heart. In addition, PPAR*α* is expressed in cells involved in immune responses including monocytes, macrophages, and lymphocytes [28]. Activation of PPAR*α* occurs through a variety of natural agonists, including unsaturated fatty acids and eicosanoids, whereas fibrate drugs act as synthetic agonists. In liver, PPAR*α* plays a pivotal role in fatty acid catabolism by upregulating the expression of numerous genes involved in mitochondrial fatty acid oxidation, peroxisomal fatty acid oxidation, and numerous other aspects of fatty acid metabolism in the cell [28]. As a consequence, activation of PPAR*α* can prevent and decrease hepatic fat storage [29–32]. Other metabolic pathways under control of PPAR*α* include gluconeogenesis [33], biotransformation [34], and cholesterol metabolism [35]. While the function of PPAR*α* in mouse liver is relatively well defined, much less is known about its role in human liver. Experiments with “humanized” PPAR*α* mice have revealed that there are intrinsic differences in the properties of the human and mouse PPAR*α* protein [36]. In general, research on the role of PPAR*α* in human liver is hampered by the low expression levels of PPAR*α* in human hepatoma cell lines [37].

Besides governing metabolic processes, PPAR*α* also regulates inflammatory processes, mainly by inhibiting inflammatory gene expression. Hepatic PPAR*α* activation has been repeatedly shown to reduce hepatic inflammation elicited by acute exposure to cytokines and other compounds. In recent years, several molecular mechanisms responsible for the immunosuppressive effects of PPAR*α* have been uncovered [38]. These include interference with several proinflammatory transcription factors including signal transducer and activator of transcription (STAT), activator protein-1 (AP-1), and NF-*κ*B by PPAR*α* [39]. The latter mechanism involves stimulation of expression of the inhibitory protein I*κ*B*α*, which retains NF-*κ*B in a nonactive state, leading to suppression of NF-*κ*B DNA-binding activity [40]. Detailed molecular studies have further revealed that PPAR*α* diminishes the activity of the proinflammatory transcription factor CAATT/enhancer binding proteins (C/EBP) via sequestration of the coactivator glucocorticoid receptor-interacting protein-1/transcriptional intermediary factor-2 (GRIP1/TIF2) [41]. Finally, PPAR*α* can also inhibit cytokine signaling pathways via downregulation of the IL-6 receptor [42] and upregulation of sIL-1 receptor antagonist [Stienstra et al., in press], leading to diminished inflammatory responses. Interestingly, in humans, specific PPAR*α* activation using fenofibrate has been shown to decrease plasma levels of several acute phase proteins that are normally increased during inflammatory conditions [42].

### 2.1. PPAR*α* and steatosis

In mice fed a high-fat diet, proper functioning of PPAR*α* is essential to prevent the liver from storing large amounts of fat [43]. By inducing mitochondrial, peroxisomal, and microsomal fatty acid oxidation, PPAR*α* reduces hepatic fat accumulation in the liver during the development of fatty liver disease, and thus prevents steatosis [31, 44, 45]. It can be hypothesized that since PPAR*α* has a potent anti-inflammatory activity in liver, the progression of steatosis towards steatohepatitis might be counteracted by PPAR*α*. Indeed, several studies in mice have shown that PPAR*α* activation is able to reduce or even reverse steatohepatitis induced by feeding a methionine- and choline-deficient (MCD) diets [31, 45, 46].

In a mouse model of steatohepatitis, the presence and activation of PPAR*α* prevented the induction of COX-2 expression [47]. Since upregulation of COX-2 is seen in alcoholic steatohepatitis and nonalcoholic steatohepatitis and has been directly linked to the progression of steatosis to steatohepatitis, the inhibitory effect of PPAR*α* on COX-2 may reduce steatohepatitis. An anti-inflammatory role of PPAR*α* in the development of steatohepatitis is further supported by a study in which wild-type and PPAR*α* −/− mice were fed a high-fat diet to induce obesity. Although both genotypes developed a fatty liver after chronic high-fat feeding, animals lacking PPAR*α* developed steatohepatitis accompanied by an increased number of infiltrated lymphocytes and macrophages. By suppressing the expression of specific chemokines involved in attracting macrophages and other immune-related cell types, PPAR*α* might moderate steatohepatitis [Stienstra et al., submitted]. These results are in line with a study performed in APOE2 knock-in mice fed a western-type high-fat diet [48]. When the animals were cotreated with fenofibrate, macrophage infiltration of the liver was prevented.

### 2.2. PPAR*α* and atherosclerosis

Inflammation in the arterial wall is known to promote the process of atherosclerosis [49]. In addition to suppressing the inflammatory response in liver, PPAR*α* may also influence inflammatory reactions in the arterial wall. As PPAR*α* is expressed in various cell types present in atherosclerotic lesions, the effect of PPAR*α* on lesion development is rather complex. Immune-modulating effects of specific PPAR*α* activation have been reported in various cell types. However, some controversy still exists about the exact role of PPAR*α* in the vascular wall as both pro- and antiatherogenic effects of PPAR*α* have been demonstrated.

An antiatherogenic effect of PPAR*α* via suppression of several proinflammatory genes like MCP-1, TNF*α*, vascular cell adhesion molecule-I (VCAM I), intercellular adhesion molecule-I (ICAM I), and interferon-*γ* (IFN*γ*) has been reported in the vascular wall of animals with extensive atherosclerosis [50]. Other studies have shown that the anti-inflammatory role of PPAR*α* in the vascular wall seems to be dependent on the severity of inflammation or vascular lesion. In the absence of inflammation or in early lesions, the effects of PPAR*α* are mainly proatherogenic [51, 52], whereas the development of severe lesions accompanied by inflammation is strongly reduced by PPAR*α* activation.

Several acute phase proteins have been linked to the development of atherosclerosis [53]. This includes CRP, which is currently used as a marker for systemic inflammation and linked to CVD, and serum amyloid A (SAA), which has been shown to be involved in the development of atherosclerosis [54]. As PPAR*α* activation downregulates plasma concentrations of acute phase proteins including CRP and SAA in humans [42], it might indirectly prevent or slow down the progression of atherosclerosis.

### 2.3. PPAR*α* and adiposity

Although expression of PPAR*α* in WAT is much lower compared to PPAR*γ*, evidence abounds that PPAR*α* may also influence adipose tissue function. It has been shown that PPAR*α* −/− mice gain more adipose mass compared to wild-type animals [55], which may be via local or systemic effects of PPAR*α*. An antiobesity role for PPAR*α* is supported by several studies in which obese rodents were administered synthetic PPAR*α* agonists [56–58]. While it is true that PPAR*α* agonists have a clear anorexic effect resulting in decreased food intake, evidence is accumulating that PPAR*α* may also directly influence adipose tissue function, including its inflammatory status.

A recent study revealed that treatment of obese diabetic KKAy mice with Wy-14643 decreased adipocyte hypertrophy as well as macrophage infiltration [59]. In PPAR*α* −/− mice chronically fed a high-fat diet (HFD), expression of inflammatory genes in adipose tissue was more pronounced compared to wild-type mice. In addition, fractionation of adipose tissue in adipocytes and stromal vascular cells revealed higher gene expression levels of the specific macrophage marker F4/80+ in the stromal vascular fraction of PPAR*α* −/− mice [Stienstra et al., submitted].

PPAR*α* may govern adipose tissue inflammation in three different ways: (1) by decreasing adipocyte hypertrophy, which is known to be connected with a higher inflammatory status of the tissue [3, 11, 59], (2) by direct regulation of inflammatory gene expression via locally expressed PPAR*α*, or (3) by systemic events likely originating from liver. Full clarification of the role of locally expressed PPAR*α* in adipose tissue will have to await the availability of adipose tissue-specific PPAR*α* −/− mice.

Thus, while evidence is mounting that PPAR*α* activation reduces adipose inflammation as observed during obesity, it is unclear whether the anti-inflammatory effects of PPAR*α* in WAT are caused by direct or indirect mechanisms.

## 3. PPAR*β*/*δ*

Compared to PPAR*α* and PPAR*γ*, much less is known about PPAR*β*/*δ* and its natural ligands. Due to its ubiquitous expression profile, lack of specific ligands and, until recently, lack of availability of knock-out models, the role of PPAR*β*/*δ* in many tissues has been poorly explored. Fortunately, the recent generation of PPAR*β*/*δ* −/− mice has provided a strong impetus for the characterization of the function of PPAR*β*/*δ* [60]. Several abnormalities have been observed in mice lacking PPAR*β*/*δ* which include impaired wound healing, a decrease in adipose mass, and disturbed inflammatory reactions in skin [61].

PPAR*β*/*δ* has been directly linked to the development of obesity. Indeed, several groups have reported a decrease in adiposity after PPAR*β*/*δ* activation. By stimulating fatty acid oxidation, PPAR*β*/*δ* activation leads to loss of adipose mass in different mouse models of obesity [62]. Similar effects on fatty acid oxidation have been observed in heart, resulting in improved muscle contraction [63]. In addition to increasing fatty acid oxidation, activation of PPAR*β*/*δ* in muscle also increases the number of type I muscle fibers, which leads to enhanced endurance performance [64].

The number of studies that have addressed the role of PPAR*β*/*δ* during inflammation is limited. So far, an anti-inflammatory effect has been observed in macrophages suggesting a possible role for PPAR*β*/*δ* in the process of atherogenic inflammation. It appears that PPAR*β*/*δ* acts as an inflammatory switch in which inactivated PPAR*β*/*δ* is proinflammatory and activated PPAR*β*/*δ* promotes an anti-inflammatory gene expression profile. The proposed switch of PPAR*β*/*δ* is linked to the B cell lymphoma-6 (BCL-6) protein which functions as inflammatory suppressor protein [65]. In the unliganded state, BCL-6 is part of the PPAR*β*/*δ*-RXR*α* transcriptional complex. Upon ligand activation, corepressors including BCL-6 are dissociated and PPAR*β*/*δ*-dependent gene transcription ensues. The released BCL-6 subsequently acts as a repressor of proinflammatory gene expression in macrophages.

### 3.1. PPAR*β*/*δ* and steatosis

It can be hypothesized that the stimulatory effect of PPAR*β*/*δ* on fatty acid oxidation in muscle and adipose tissue might also extend to liver, which would render PPAR*β*/*δ* an antisteatotic role in liver. Within the liver, PPAR*β*/*δ* expression is found in different cell types although the highest levels are found in hepatic endothelial cells [66].

According to a recent report by Nagasawa et al., activation of PPAR*β*/*δ* may diminish fatty liver disease. In this study, mice were fed an MCD diet to induce steatohepatitis. Administration of the PPAR*β*/*δ* agonist GW501516 not only decreased hepatic lipid content, yet it also reduced inflammatory gene expression. PPAR*β*/*δ* decreased fat storage in liver mainly by activation of genes involved in fatty acid oxidation. Furthermore, the elevated mRNA levels of transforming growth factor-*β*1 (TGF-*β*1), TNF*α*, MCP-1 and interleukin-1*β* (IL-1*β*) that accompany the development of steatohepatitis were counteracted by PPAR*β*/*δ* activation [67]. Which liver cell types and molecular mechanisms contribute to the observed regulation is unknown.

### 3.2. PPAR*β*/*δ* and atherosclerosis

Due to the anti-inflammatory properties of PPAR*β*/*δ* in macrophages, it is plausible that atherosclerosis is affected by PPAR*β*/*δ*-activation. By feeding low-density lipoprotein receptor (LDLR) −/− mice a hypercholesterolemic diet supplemented with a specific PPAR*β*/*δ* ligand, it was shown that PPAR*β*/*δ* is able to interfere with the inflammatory process underlying the development of atherosclerosis. Whereas lesion development itself was not prevented by PPAR*β*/*δ* activation, inflammatory gene expression was blunted compared to untreated mice [50]. The anti-inflammatory action of PPAR*β*/*δ* was mainly achieved by a strong inhibition of VCAM-1, MCP-1, and IFN-*γ* expressions, genes that are associated with the development of atherosclerosis. A recent study in which LDLR −/− mice were treated with the PPAR*β*/*δ* agonist GW0742X revealed an antiatherosclerotic effect of PPAR*β*/*δ*, in addition to an anti-inflammatory effect. Lesion development was strongly inhibited and inflammatory gene expression in macrophages was decreased [68].

While in mice there is compelling evidence for an anti-inflammatory role of PPAR*β*/*δ* in the atherosclerosis, the role of PPAR*β*/*δ* in humans is relatively unknown. Remarkably, PPAR*β*/*δ* was shown to strongly promote lipid accumulation in human macrophages, thereby supporting the development of atherosclerosis [69]. Whether PPAR*β*/*δ* influences inflammatory gene expression in human cells needs further study.

### 3.3. PPAR*β*/*δ* and adiposity

Recently, it was shown that activation of PPAR*β*/*δ* in adipose tissue causes a marked decrease in fat mass which is mainly achieved by activation of fatty acid oxidative pathways [62]. Moreover, high-fat-diet-induced adiposity was strongly inhibited by activation of PPAR*β*/*δ* in adipose tissue. Whether PPAR*β*/*δ* is able to control inflammatory gene expression in WAT during diet-induced obesity is still unclear. Inasmuch as inflammatory gene expression is linked to adiposity, it could be hypothesized that inflammatory gene expression will be suppressed by PPAR*β*/*δ* activation. Also, since expressions of IL-1*β*, MCP-1, and TNF*α* are controlled by PPAR*β*/*δ* in liver [67], it is tempting to speculate that inflammatory gene expression is under control of PPAR*β*/*δ* in adipose tissue as well.

## 4. PPAR*γ*

PPAR*γ* is considered the master regulator of adipogenesis, and accordingly has been extensively studied in the context of obesity. In humans, PPAR*γ* is most highly expressed in adipose tissue, yet reasonable levels of PPAR*γ* mRNA can also be found in other organs including skeletal muscle, colon, and especially lung [70]. The latter is probably due to the abundance of macrophages in lung. At least two different isoforms of PPAR*γ* are known: PPAR*γ*1, which is the form expressed in nonadipose tissues, and PPAR*γ*2, which is adipose-tissue specific. Unsaturated fatty acids and several eicosanoids serve as endogenous agonists of PPAR*γ*, while antidiabetic drugs belonging to the thiazolidinediones act as synthetic agonists of PPAR*γ*. Target genes of PPAR*γ* are involved in adipocyte differentiation, lipid storage, and glucose metabolism, and include lipoprotein lipase, CD36, phosphoenolpyruvate carboxykinase, aquaporin 7, and adiponectin [71].

Gain and loss of function studies have shed more light on the specific functions of PPAR*γ* in different tissues. While homozygous PPAR*γ*-deficient animals are embryonically lethal, specific ablation in adipose tissue revealed the indispensable role of PPAR*γ* in adipocyte differentiation and function [72]. In liver, PPAR*γ* is involved in triglyceride homeostasis and contributes to steatosis. At the same time, hepatic PPAR*γ* protects other tissues from triglyceride accumulation and insulin resistance [73].

Similar to PPAR*α*, PPAR*γ* is involved in governing the inflammatory response, especially in macrophages. Currently, two different molecular mechanisms have been proposed by which anti-inflammatory actions of PPAR*γ* are effectuated: (1) via interference with proinflammatory transcription factors including STAT, NF-*κ*B, and AP-1 [74], and (2) by preventing removal of corepressor complexes from gene promoter regions resulting in suppression of inflammatory gene transcription [75]. This mechanism involves ligand-dependent SUMOylation of PPAR*γ* followed by binding of PPAR*γ* to nuclear receptor corepressor (NCoR)-histone deacetylase-3 (HDAC3) complexes localized on inflammatory gene promoters. The binding of PPAR*γ* prevents the removal of corepressor complexes, thus retaining inflammatory genes in a suppressed state.

### 4.1. PPAR*γ* and adiposity

PPAR*γ* is indispensable for adipocyte differentiation both in vivo and in vitro [76–78]. In spite of its vital role in adipogenesis and lipogenesis, PPAR*γ* expression itself is not strongly influenced during obesity. As discussed above, diet-induced obesity is associated with increased inflammatory gene expression in adipose tissue via adipocyte hypertrophy and macrophage infiltration. It has been shown that PPAR*γ* is able to reverse macrophage infiltration, and subsequently reduces inflammatory gene expression [18]. Adipose expression of inflammatory markers A disintegrin and metallopeptidase domain-8 (ADAM8), macrophage inflammatory protein-1*α* (MIP-1*α*), macrophage antigen-1 (MAC-1), F4/80+, and CD68 was downregulated by specific PPAR*γ* activation. Inflammatory adipokines mainly originate from macrophages which are part of the stromal vascular fraction of adipose tissue [18, 19], and accordingly, the downregulation of inflammatory adipokines in WAT by PPAR*γ* probably occurs via effects on macrophages. By interfering with NF-*κ*B signaling pathways, PPAR*γ* is known to decrease inflammation in activated macrophages [74]. PPAR*γ* may also influence inflammatory gene expression via effects on adipocyte morphology. Indeed, smaller adipocytes are known to secrete less inflammatory markers compared to larger adipocytes [3]. Treatment of obese rats with the synthetic PPAR*γ* agonist troglitazone dramatically reduced the size of adipocytes without changing the total weight of WAT. In parallel, the expression levels of the inflammatory marker TNF*α* were normalized compared to those of untreated rats [79]. Furthermore, by inducing the expression of adiponectin in adipocytes [80], PPAR*γ* may directly contribute to suppression of chronic inflammation accompanying obesity.

Summarizing, the anti-inflammatory effects of PPAR*γ* activation in adipose tissue are presumably achieved by effects on both adipocytes and adipose tissue-resident macrophages. Interestingly, PPAR*γ* is induced both during macrophage and adipocyte differentiation [71]. Since preadipocytes that are present in adipose tissue have the ability to differentiate towards macrophage-type cells and towards adipocytes depending on the local environment [81], the role of PPAR*γ* in determining the fate of preadipocytes is of interest. It can be hypothesized that activation of PPAR*γ* might favor adipocyte differentiation resulting in a decreased inflammatory status of adipose tissue during obesity.

### 4.2. PPAR*γ* and atheroslerosis

PPAR*γ* is expressed in white blood cells and differentiated macrophages and has been implicated in the process of atherosclerosis. Initially, PPAR*γ* activation was proposed to be proatherogenic by stimulating uptake and storage of oxidized lipids in macrophages via upregulation of the scavenger receptor/fatty acid transporter CD36. This process leads to foam cell development and is a key event in the development of atherosclerosis [82]. In contrast, treatment with thiazolidinediones has been shown to reduce the development of atherosclerosis in mouse models [50, 71], suggesting that PPAR*γ* is antiatherogenic. The inhibitory effect on atherosclerosis may be mediated by upregulating expression of the ABCA1 transporter in macrophages, thereby promoting cholesterol efflux. Furthermore, PPAR*γ* activation strongly reduces inflammatory gene expression in macrophages, including MCP-1, VCAM-1, ICAM-1, IFN*γ*, and TNF*α* [50]. Several human studies also point to antiatherogenic effects of PPAR*γ* in type II diabetic patients. Daily administration of 400 mg troglitazone or 30 mg pioglitazone for 6 months resulted in a reduction of common carotid arterial intimal and medial complex thickness which is used as a noninvasive method to monitor early atherosclerotic lesions [83, 84]. In a randomized controlled trial using 5238 patients with type II diabetes, treatment with 15 mg to 45 mg pioglitazone improved cardiovascular outcome [85]. Whether these protective effects in humans are achieved by inhibiting inflammation remains to be determined.

### 4.3. PPAR*γ* and steatosis

It has been well established that in mouse models of steatosis, the development of fatty liver is associated with increased hepatic expression of PPAR*γ*. In a nonfatty liver, the role of PPAR*γ* appears to be limited and is probably restricted to stellate cell function during liver injury-induced fibrogenesis [86]. During the development of steatosis, hepatocytes become lipid-loaden and gain phenotypical characteristics of adipocytes which include the formation of large lipid droplets. In parallel, expression of adipogenic and lipogenic genes such as sterol regulatory element binding protein (SREBP), Adipose differentiation-related protein (ADRP) and PPAR*γ* are strongly upregulated in steatotic livers [87, 88]. Likely, the upregulation of PPAR*γ* contributes to the phenotype, since adenoviral-mediated hepatic overexpression of PPAR*γ*1 on a PPAR*α* −/− background dramatically increases hepatic lipid accumulation and adipogenic gene expression in mice [89]. Also, marked upregulation of PPAR*γ* in livers of PPAR*α* −/− mice fed a high-fat diet leads to increased expression of adipocyte markers and might contribute to the fatty liver phenotype [43]. In contrast, mice that specifically lack PPAR*γ* in liver are protected from hepatic steatosis and show decreased expression levels of lipogenic genes compared to wild-type mice [73, 90]. Thus, PPAR*γ* induction appears to be necessary and sufficient for hepatic steatosis.

The development of steatosis and progression into steatohepatitis is closely linked to an increased inflammatory state of the liver [4]. Recent data suggest that activation of PPAR*γ* in fatty liver may protect against inflammation. Microarray analysis revealed that several inflammatory genes that are upregulated in fatty livers of mice fed a high-fat diet were strongly downregulated by PPAR*γ* overexpression in liver [89]. These genes include SAA, Chemokine (C-X-C motif) ligand 10 (CXL10)/IP10 and interferon-*γ*-inducible protein, 47 kd. Data from our own group showed that hepatic PPAR*γ* activation by rosiglitazone under steatotic conditions results in downregulation of multiple proinflammatory genes. Thus, although activation of PPAR*γ* in liver contributes to the development of steatosis, inflammatory gene expression is suppressed.

Several small clinical human studies have been performed to evaluate the effects of thiazolidinediones in patients diagnosed with NASH. After treatment, the degree of steatosis and inflammation improved in a number of patients indicating that PPAR*γ* may be an interesting pharmacological target [91]. Apart from weight gain, no side effects were reported in these studies. However, more studies are needed to assess the potentially beneficial effects of PPAR*γ* activation on liver function.

## 5. CONCLUSION

**Figure 1:**
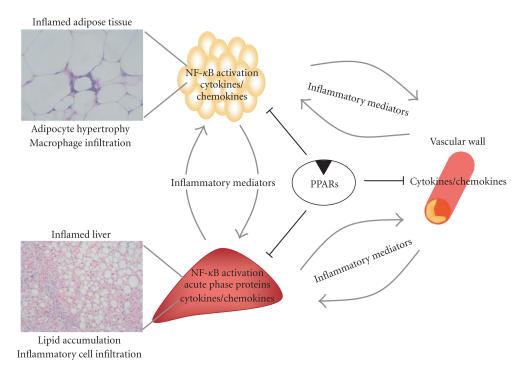
Central role of PPARs in obesity-induced inflammation. (Visceral) obesity and associated fatty liver stimulate inflammation in adipose tissue and liver via increased recruitment and infiltration of macrophages, resulting in increased production of proinflammatory cytokines. By downregulating proinflammatory genes in liver, adipose tissue and the vascular wall, PPARs have a major influence on the progression of obesity-related inflammation and its complications.

An elevated inflammatory status is increasingly believed to be an important mediator that links excess (visceral) fat mass with numerous metabolic abnormalities, including insulin resistance. PPARs may influence the inflammatory response either by direct transcriptional downregulation of proinflammatory genes via mechanisms involving transrepression, or indirectly via their transcriptional effects on lipid metabolism. Numerous animal studies have demonstrated a role for PPARs in counteracting obesity-induced inflammation in liver, adipose tissue, and the vascular wall. The ability to reduce inflammatory cell infiltration further underlines the central role of PPARs in obesity-induced inflammation (Figure 1).

A growing number of studies strongly support anti-inflammatory properties of PPARs in human obesity as well. Several clinical trials in type II diabetic or hyperlipidemic patients have clearly shown that PPAR*α* agonists including fenofibrate, ciprofibrate, and gemfibrozil can effectively reduce circulating levels of TNF*α*, IL-6, fibrinogen, and CRP [92]. Rosiglitazone, a selective PPAR*γ* agonist, exerts anti-inflammatory effects in both obese and type II diabetic individuals by decreasing plasma concentrations of C-reactive protein, serum amyloid-A, and matrix metalloproteinase [93, 94].

Since synthetic PPAR*α* and PPAR*γ* agonists independently ameliorate obesity-induced inflammation, agonists that activate both PPAR*α* and PPAR*γ* (the so-called dual PPAR*α*/PPAR*γ* agonists) might be even more effective. Unfortunately, the development and clinical trials of these compounds have been hampered by serious concerns regarding their safety. Many dual PPAR*α*/PPAR*γ* agonists once in clinical development have since been abandoned, often for reasons of toxicity, including most recently the dual agonist tesaglitazar.

In conclusion, although more work is needed to evaluate their full potential in humans, especially in terms of safety, PPAR agonists nevertheless represent a promising strategy to mitigate obesity-associated inflammation.
